# P-2032. Monitoring the laboratory frequency for musculoskeletal infections at a tertiary care Children's Hospital: a diagnostic stewardship QI project

**DOI:** 10.1093/ofid/ofaf695.2196

**Published:** 2026-01-11

**Authors:** Kedar Tilak, Christine A Symes, Douglas S Swanson, Rana E El Feghaly, Alaina N Burns, Ann Wirtz

**Affiliations:** Children's Mercy Hospital, Overland Park, KS; Children's Mercy Hospitals and Clinics, Kansas City, Missouri; Children's Mercy, Kansas City, Missouri; Children's Mercy Kansas City, Kansas City, MO; Children's Mercy Kansas City, Kansas City, MO; Children's Mercy Kansas City, Kansas City, MO

## Abstract

**Background:**

Patients with musculoskeletal infections (MSKI) undergo several laboratory tests. To promote laboratory stewardship, our Infectious diseases (ID) division recommended the following frequencies for labs: 1) C-reactive protein (CRP) every 2-3 days until >50% reduction while >3mg/dL, otherwise weekly until normal, 2) Sedimentation rate (ESR) at the start of therapy and end if > 20mm/hr initially, 3) Complete blood cell count (CBC) at the start of therapy and when transitioning to oral therapy if WBC is initially elevated. Baseline data showed that of 42 children evaluated between July 2023-May 2024, only 15 (35.7%) had a CBC and 13 (30.95%) had a CRP and ESR obtained at the recommended frequency. Our aim was to increase appropriately ordered monitoring labs to 65% by October 2025.

**Figure:**
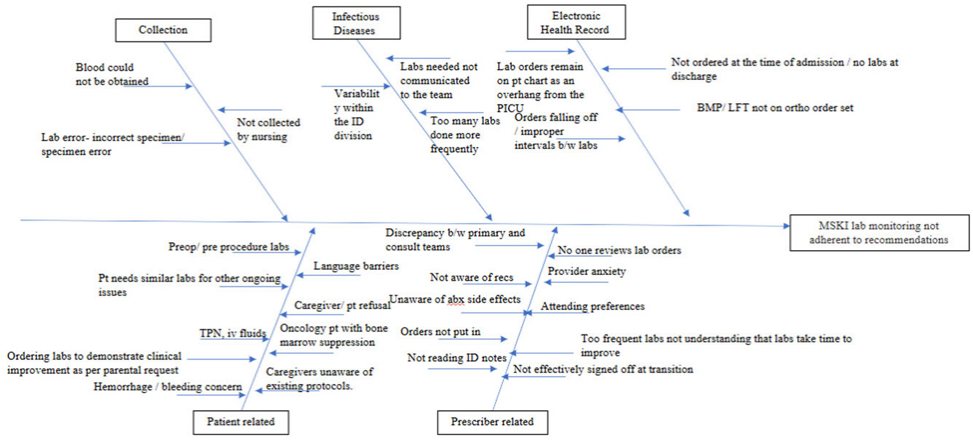
Cause and effect analysis

**Figure:**
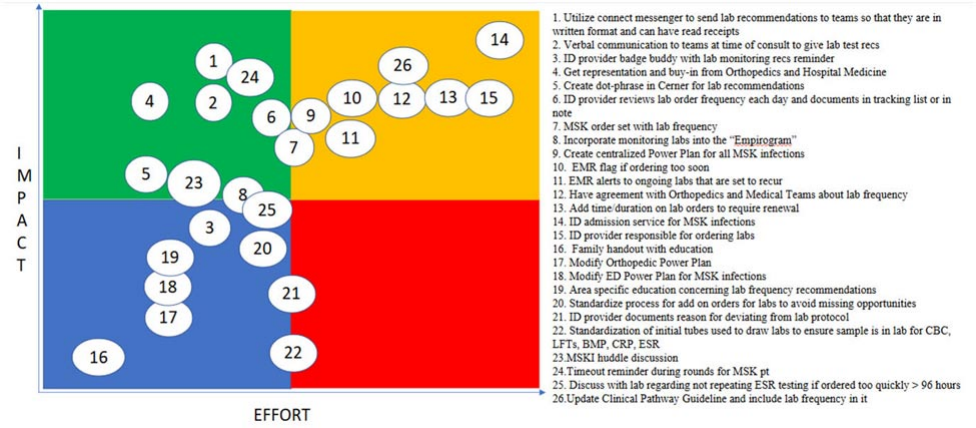
PICK chart

**Methods:**

We formed a quality improvement team (QI) of ID specialists (fellow, advanced practice provider, pharmacists, physician) and consulted with orthopedics and hospital medicine physicians. We followed QI methodology to create a cause-and-effect analysis (Figure 1) and prioritization matrix (Figure 2). Our Plan-Do-Study-Act (PDSA) cycles included: 1) An electronic health record (EHR) template to use in notes and emphasizing verbal communication with team; 2) Daily huddle discussion of MSKI and performing time-out before entering patients’ rooms. Our outcome measures were the percentage of patients with MSKI having laboratory frequency in accordance with our consensus, our process measure was the percentage of time the laboratory frequency is recommended in the ID consultation note, and our balancing measure was hospital readmission rate for patients with MSKI. We used control charts to display data and followed Shewhart rules to shift center lines.

Annotated control charts of the percentage of encounters with appropriate laboratory tests A.ESR; B.CRP; C.CBC

**Figure:**
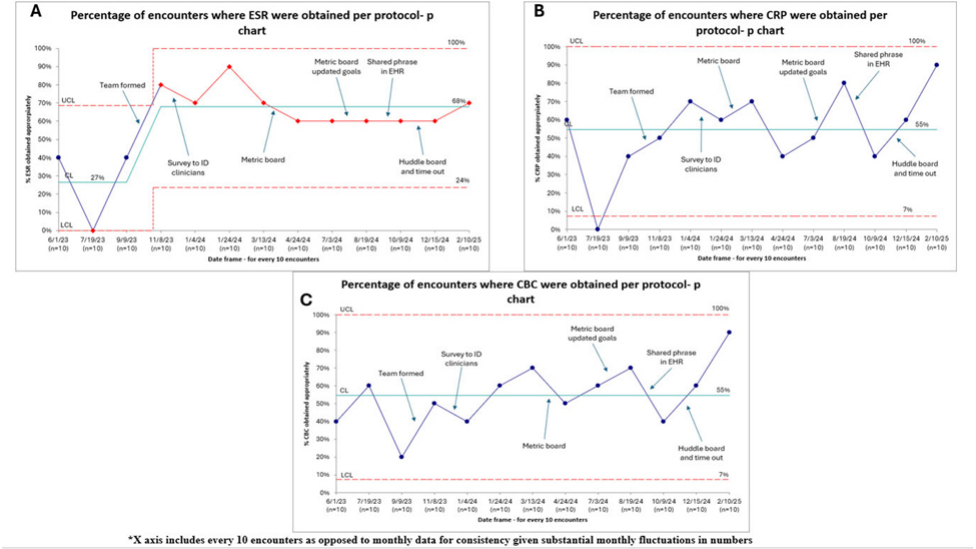

**Results:**

We saw a shift in appropriate ESR frequency testing in November 2023 from 27% to 68% shortly after the team formed (Figure 3 A). We have not observed a shift in the frequency of CBC or CRP (Figure 3B, 3C). We saw an increase in our process measure from 0% to 60%. We saw no change in our balancing measure.

**Conclusion:**

Our QI initiative aimed at enhancing the frequency of laboratory monitoring for patients with MSKI has shown promising trends. Continued efforts and adjustments to our strategies are essential to achieve our goal of 65% by October 2025.

**Disclosures:**

Rana E. El Feghaly, MD, MSCI, CPHQ, Merck (Any division): Grant/Research Support|Pfizer: Honoraria|Pfizer: Grant review panel

